# Real‐life data on inactivated COVID‐19 vaccination in patients with subcutaneous allergen immunotherapy

**DOI:** 10.1002/clt2.12115

**Published:** 2022-01-20

**Authors:** Yingyang Xu, Kai Guan

**Affiliations:** ^1^ Department of Allergy, Peking Union Medical College Hospital Chinese Academy of Medical Sciences & Peking Union Medical College Beijing China; ^2^ Beijing Key Laboratory of Precision Medicine for Diagnosis and Treatment on Allergic Diseases Beijing China; ^3^ National Clinical Research Center for Dermatologic and Immunologic Diseases Beijing China

**Keywords:** allergen immunotherapy, COVID‐19 vaccination, subcutaneous immunotherapy, adverse effects

1

To the Editor,

Allergen‐specific immunotherapy (AIT) is an important therapeutic option for allergic diseases mediated by IgE. Subcutaneous immunotherapy (SCIT) requires patients to regularly visit their doctors and receive injections at weekly or monthly intervals for at least 3 years. However, the ongoing COVID‐19 pandemic has influenced the routine for SCIT and impacted adherence to SCIT to a large degree.^1^, ^2^

Vaccination is considered as an effective strategy to prevent COVID‐19 infection and ameliorate its outcome. Thus far, two mRNA‐based vaccines, three inactivated‐virus vaccines and two adenovirus‐vector vaccines have mainly been used in countries worldwide. COVID‐19 vaccines are promoted to general people and more than 78 billion vaccine doses have been administered globally at the time of writing this paper. This situation raises new concerns about the relationship between COVID‐19 vaccination and SCIT for both allergists and patients undergoing SCIT. Although the European Academy of Allergy and Clinical Immunology recommended that AIT should be separated from vaccination for infectious diseases by at least 1 week,^3^ this is not the case in clinical practice. In fact, the interval between AIT and COVID‐19 vaccination recommended by physicians and even some allergists is 1–4 weeks or even longer. Such long intervals will impact the regular injections for SCIT, especially during the up‐dosing phase. Thus, determination of an appropriate interval that can balance the safety of the injections and minimize the influence on SCIT is needed. Unfortunately, real‐life data on this topic are inadequate. This led us to explore the safety of simultaneous receipt of COVID‐19 vaccination and SCIT.

A web‐based survey was conducted from 21 June to 23 July 2021. Patients who received regular SCIT in the Allergy Department at Peking Union Medical College Hospital (PUMCH) were invited to participate in an electronic questionnaire (Table 1) through the Wenjuanxing platform on social media. The study was approved by the Research and Ethics Board of PUMCH (S‐K1668).

**TABLE 1:** Data on COVID‐19 vaccination in patients undergoing subcutaneous immunotherapy

General information	Number	Percentage
Total number of patients completed questionnaire (male)	222 (103)	
Age (median, years)	19–65 (35)	
Diagnosis		
Allergic rhinoconjunctivitis	141	
Asthma	10	
Allergic rhinoconjunctivitis and asthma	67	
Atopic dermatitis	20	
Other complication (urticaria, anaphylaxis, drug allergy, food allergy)	54	
Vaccination information		
Number of patients receiving SARS‐CoA vaccination	143	64%
Number of patient unreceiving SARS‐CoA vaccination	79	36%
In vaccinated patients		
Feeling anxiety about the negative interference between SCIT and vaccination	57	40%
Consulting doctor before vaccinated	97	68%
Stop SCIT before and after vaccination	66	46%
Interval between SCIT and COVID‐19 vaccination		
1 week respectively	56	39%
2 weeks respectively	7	5%
At least 4 weeks respectively	3	2%
Self‐report vaccination related adverse reactions		
Local reactions		
Pain, swelling, redness, pruritus or induration of injection site	9	6%
Systemic reactions		
Fatigue	10	7%
Headache	7	5%
Fever	5	3%
Drowsiness	5	3%
Rash	4	3%
Dizziness	4	3%
Cough	3	2%
Nausea	3	2%
Vomiting	2	1%
Decreased appetite	2	1%
Palpitation	2	1%
Diarrhea	1	1%
Anaphylaxis	0	0%
Anaphylactoid purpura	0	0%
Chest pain	0	0%
Visiting doctors or use any drug to treat the vaccination related adverse reactions	3	2%
Feeling allergic disease aggravate after vaccination	4	3%
Observed SCIT adverse reaction during vaccination	0	0%

Abbreviation: SCIT, subcutaneous immunotherapy.

A total of 222 patients with SCIT completed the questionnaire. Of these, 143 (64%) received two doses of inactivated COVID‐19 vaccine (Corona Vac [Sinovac, China] or BIBBP‐CorV [Sinopharm, China]) during their SCIT schedule.

Among the vaccinated patients, 57 (40%) had anxiety about negative interference between their vaccine and SCIT/allergic disease. Ninety‐seven (68%) patients consulted an allergist about whether they could be vaccinated and whether an intermission for SCIT was needed during vaccination. As a result, 66 (46%) patients stopped SCIT during vaccination, of whom 56 patients stopped SCIT for 1 week before and after vaccination as recommended by an allergist based on the guidelines.^3^, ^4^ For the remaining patients, the interval between SCIT and vaccination was 2 weeks in seven patients and at least 4 weeks in three patients. Seventy‐seven (54%) patients did not change their SCIT schedule during vaccination, but only five patients were administered the two types of injections simultaneously because they did not inform doctors that they had received another injection on the same day. The findings for the patients were similar to the results of an international survey in the allergy community.^5^ In that survey, 58% of doctors would not change the AIT schedule and 77% of doctors would not stop AIT before vaccination.^5^ A total of 27 (18%) patients reported vaccination‐related adverse reactions. Local adverse reactions at the injection site, including pain, swelling, redness, or pruritus, were reported by 9 (6%) patients. Fatigue (7%) was the most common systemic reaction, followed by headache (5%), fever (3%), drowsiness (3%), rash (3%), dizziness (3%), cough (2%), nausea (2%), vomiting (1%), decreased appetite (1%), palpitation (1%), and diarrhea (1%). Most of these adverse reactions were mild, and only three patients received treatment. Compared with clinical trials of COVID‐19 vaccines,^6^ headache (1%–3% vs. 5%), drowsiness (0%–1% vs. 3%), and dizziness (0%–1% vs. 3%) seemed to be higher in the present study, while other adverse reactions were similar to those in the clinical trials. No previously reported severe adverse reactions, such as anaphylaxis, cardiac injury, and thrombotic thrombocytopenia,^6^, ^7^, ^8^ were observed in the present study. The impact of SCIT interruption on adverse reactions to COVID‐19 vaccines was further analyzed. No difference in adverse reactions to COVID‐19 vaccines between patients who stopped and did not stop SCIT was found by the chi‐square test and no correlation between SCIT interruption and adverse reactions to vaccines was found by the Spearman correlation test.

In a recent survey conducted on 56 doctors with experience of AIT management, 77% did not stop AIT before vaccination and 70% considered that COVID‐19 vaccines were similar to vaccines for other infectious diseases.^5^ It has been reported that no systemic reactions were observed in patients who simultaneously received vaccines for other infectious diseases and SCIT.^9^ Our findings provide real‐life data on adverse reactions to COVID‐19 vaccines in patients receiving SCIT and support that the notion that patients receiving SCIT do not exhibit increased adverse reactions to COVID‐19 vaccines compared with the general population. Furthermore, interruption or continuation of SCIT does not affect the occurrence of adverse reactions to COVID‐19 vaccines.

To summarize, the safety of inactivated COVID‐19 vaccines in patients undergoing SCIT is similar to that in the general population and patients without interruption of SCIT during COVID‐19 vaccination do not exhibit increased adverse reactions to the vaccines.

## CONFLICT OF INTEREST

None of the authors has potential conflict of interest related to this manuscript.

## AUTHOR CONTRIBUTIONS

Yingyang Xu has made substantial contributions to design of the study, acquisition of data, analysis and interpretation of data, and drafting the article. Kai Guan has made substantial contributions to design of the study, acquisition of data, and giving final approval of the version to be submitted.

## References

[1] Pfaar O , Agache I , Bonini M , et al. COVID‐19 pandemic and allergen immunotherapy—an EAACI survey. Allergy. 2021;76:3504‐3516. 33655519 10.1111/all.14793 PMC8013670

[2] Kulhas Celik I , Metbulut AP , Uneri OS , Senses Dinc G , Dibek Misirlioglu E . Effect of patient and parental anxiety on adherence to subcutaneous allergen immunotherapy during the coronavirus disease 2019 pandemic. Ann Allergy Asthma Immunol. 2021;126:595‐597. 33493638 10.1016/j.anai.2021.01.025 PMC7825879

[3] Alvarez‐Cuesta E , Bousquet J , Canonica GW , Durham SR , Malling HJ , Valovirta E . Standards for practical allergen‐specific immunotherapy. Allergy. 2006;61(Suppl 82):1‐3. 10.1111/j.1398-9995.2006.01219_1.x 16930249

[4] Klimek L , Pfaar O , Hamelmann E , et al. COVID‐19 vaccination and allergen immunotherapy (AIT)—a position paper of the German Society for Applied Allergology (AeDA) and the German Society for Allergology and Clinical Immunology (DGAKI). Allergol Select. 2021;5:251‐259. 34533543 10.5414/ALX02245E PMC8439106

[5] Masieri S , Bachert C , Ojeda P , Kim C‐K , Carlo C , Giorgio C . Allergen immunotherapy management during vaccinations: an international survey. World Allergy Organ J. 2021;14:100601. 34777679 10.1016/j.waojou.2021.100601 PMC8575483

[6] Zhang Y , Zeng G , Pan H , et al. Safety, tolerability, and immunogenicity of an inactivated SARS‐CoV‐2 vaccine in healthy adults aged 18–59 years: a randomised, double‐blind, placebo‐controlled, phase 1/2 clinical trial. Lancet Infect Dis. 2021;21:181‐192. 33217362 10.1016/S1473-3099(20)30843-4 PMC7832443

[7] Rzymski P , Perek B , Flisiak R . Thrombotic thrombocytopenia after COVID‐19 vaccination: in search of the underlying mechanism. Vaccines (Basel). 2021;9:559. 34071883 10.3390/vaccines9060559 PMC8227748

[8] Li M , Yuan J , Lv G , Brown J , Jiang X , Lu ZK . Myocarditis and pericarditis following COVID‐19 vaccination: inequalities in age and vaccine types. J Pers Med. 2021;11:1106. 34834458 10.3390/jpm11111106 PMC8624452

[9] Ullrich D , Ullrich K , Mussler S , Thum‐Oltmer S . Vaccination during concurrent subcutaneous immunotherapy: safety of simultaneous application. Eur Ann Allergy Clin Immunol. 2015;47(1):10‐14. 25599553

